# High-resolution mapping and characterization of *qRgls2*, a major quantitative trait locus involved in maize resistance to gray leaf spot

**DOI:** 10.1186/s12870-014-0230-6

**Published:** 2014-08-31

**Authors:** Ling Xu, Yan Zhang, Siquan Shao, Wei Chen, Jing Tan, Mang Zhu, Tao Zhong, Xingming Fan, Mingliang Xu

**Affiliations:** National Maize Improvement Center of China, China Agricultural University, No.2 Yuanmingyuan West Road, Beijing, 100193 PR China; Baoshan Institute of Agricultural Science, Taibao North Road, Longyang District, Baoshan, 678000 PR China; Institute of Food Crops, Yunnan Academy of Agricultural Sciences, Longtou Street, Kunming, 650205 PR China

**Keywords:** Maize, GLS, QTL, Fine-mapping, Candidate genes

## Abstract

**Background:**

Gray leaf spot (GLS) caused by *Cercospora zeae-maydis* (*Czm*) or *Cercospora zeina* (*Cz*) is a devastating maize disease and results in substantial yield reductions worldwide. GLS resistance is a quantitatively inherited trait. The development and cultivation of GLS-resistant maize hybrids are the most cost-effective and efficient ways to control this disease.

**Results:**

We previously detected a major GLS resistance QTL, *qRgls2*, in bin 5.03–04, which spans the whole centromere of chromosome 5 encompassing a physical distance of ~110-Mb. With advanced backcross populations derived from the cross between the resistant Y32 and susceptible Q11 inbred lines, a sequential recombinant-derived progeny testing strategy was adapted to fine map *qRgls2*. We narrowed the region of *qRgls2* from an initial ~110-Mb to an interval of ~1-Mb, flanked by the markers G346 and DD11. *qRgls2* showed predominantly additive genetic effects and significantly increased the resistance percentage by 20.6 to 24.6% across multiple generations. A total of 15 genes were predicted in the mapped region according to the 5b.60 annotation of the maize B73 genome v2. Two pieces of the mapped *qRgls2* region shared collinearity with two distant segments on maize chromosome 4.

**Conclusions:**

*qRgls2*, a major QTL involved in GLS resistance, was mapped to a ~1-Mb region close to the centromere of chromosome 5. There are 15 predicted genes in the mapped region. It is assumed that *qRgls2* could be widely used to improve maize resistance to GLS.

**Electronic supplementary material:**

The online version of this article (doi:10.1186/s12870-014-0230-6) contains supplementary material, which is available to authorized users.

## Background

Gray leaf spot (GLS) is a destructive fungal disease and poses a serious threat to maize production worldwide. The yield loss caused by GLS varies with different environmental conditions and cultivars. For instance, epidemic GLS could result in >50% yield loss in the United States [1], 20–50% in Brazilian Central Region [2], and 20 to 60% in South Africa [3].

Both *Cercospora zeae-maydis* (*Czm*) and *Cercospora zeina* (*Cz*) are considered to be the casual pathogens to GLS [4]. *Cercospora* spores overwinter on corn debris left in the field, until conidia begin to develop in warm temperature and high humidity in the next growing season [5,6]. After initial pathogen infection, GLS lesions appear first on the bottom leaves. In the early stages, it is hard to distinguish GLS symptoms from those of other foliar diseases, such as Northern corn leaf blight and Southern corn leaf blight. Some mature GLS lesions, however, have unique features and are characterized by their distinct rectangular shapes parallel to the veins [7].

Compared with regular methods for disease control (fungicide spraying, conventional tillage, and intercropping), cultivation of GLS-resistant hybrids is a cost-effective and environmentally friendly way to reduce yield loss due to gray leaf spot [8]. GLS resistance is a canonical quantitatively-inherited trait [9]. Moreover, general combining ability is predominant in GLS resistance, implying that additive genetic effects are more important than non-additive effects for resistance development [10-12]. Thus, QTL identification would accelerate the breeding of resistant hybrids. For example, the simple sequence repeat (SSR) markers linked to QTLs in bins 4.03 and 4.04 were used to select GLS-resistant maize [13]. So far, a quite number of QTLs associated with GLS resistance have been reported using various parental lines and mapping groups [7,14-19]. Out of 57 resistance QTLs, seven consensus QTLs were found on chromosome bins 1.06, 2.06, 3.04, 4.06, 4.08, 5.03, and 8.06 [20]. QTLs for GLS resistance were reported to show stable genetic contributions to GLS resistance in different environments [2]. Some resistance QTLs were localized in the region associated with multiple-disease resistance, suggesting an intriguing broad-spectrum resistance [21]. In our previous study, we used the GLS-resistant line Y32 and the GLS-susceptible line Q11 to develop mapping populations for QTL analysis. One of the major QTLs in bin 8.01–03 is restricted to an ~1.4-Mb region. Another major QTL, *qRgls2* in bin 5.03–04, is located within an ~110-Mb region spanning the whole centromere of chromosome 5 [22].

Using a recombinant-derived progeny testing strategy, we ultimately mapped *qRgls2* to a ~1-Mb interval on chromosome 5, which was close to the heterochromatin portion around the centromere. The mapped *qRgls2* region shows syteny with two distant segments on chr.4 in maize as well as one segment on chr.2 in rice. Our results provide useful information for *qRgls2* cloning, and the markers developed around *qRgls2* can be readily used for breeding GLS-resistant maize.

## Methods

### Plant materials

**Figure 1:**
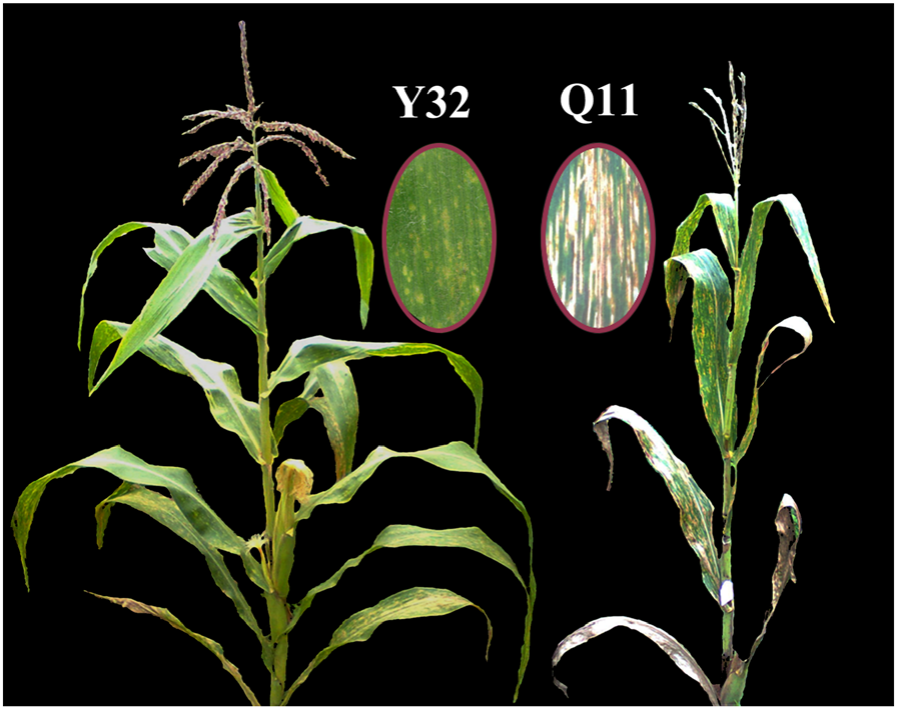
**GLS symptoms of resistant line Y32 (left) and susceptible line Q11 (right).** The ovals show enlarged images of the leaves. Y32 had a disease scale of 3; Q11 had a disease scale of 7.

A highly GLS-resistant inbred line, Y32, was developed from the tropical population Suwan1 and used as a donor parent. The highly GLS-susceptible line Q11 was used as a recurrent receptor parent (Figure 1). The two parental lines were crossed to produce the F_1_ hybrid, which was continuously selfed to generate F_2_, F_2:3_, and F_3:4_ populations. The F_1_ and F_2_ populations were planted in Kunming (Yunnan province, China). The 161 F_2:3_ families derived from 161 F_2_ individuals were evaluated for GLS resistance in Baoshan and Dehong (Yunnan province, China). In the initial QTL mapping, the mean disease scale of each F_2:3_ family was used to describe the disease state of the parental F_2_ individual [22]. In the winter nursery of 2010/2011, the F_3:4_ families was planted in Jinghong (Yunnan province, China) to screen for recombinants using flanking markers bnlg1046 and umc1171. Then, recombinants were identified and backcrossed to Q11 to produce BC_1_F_4_ progeny, which were planted in Baoshan to evaluate GLS disease severity. We planted the BC_1_F_4_ progeny of each F_3:4_ recombinant into one plot and all plots were randomly distributed. Every plot has eight rows, 3.5 m in length and 0.5 m in width each row. Each row has 15 holes. Totally, 120 seeds were sowed per plot. Because of the severity of GLS in the recurrent parent Q11, it was impossible to backcross BC_1_F_4_ individuals to Q11. Therefore, we selfed each BC_1_F_4_ individual as well as the BC_2_F_5_ and BC_3_F_6_ populations in Baoshan. In 2011/2012, we planted BC_1_F_4:5_ families in the winter nursery in Jinghong to screen for more recombinants. The resultant recombinants were then backcrossed to Q11 to develop BC_2_F_5_ progeny. The BC_2_F_5_ population was grown in Boshan to investigate its GLS resistance. The BC_2_F_5_ progeny of each BC_1_F_4:5_ recombinants were planted in one plot with ten rows. There are 16 holes each row. Totally, 160 seeds were sowed per plot. In 2012/2013, the BC_2_F_5:6_ families were grown in Sanya (Hainan province, China) to obtain recombinants. In 2013, BC_3_F_6_ plants derived from BC_2_F_5:6_ recombinants were grown in Boshan for fine-mapping of *qRgls2*. We arranged 12 rows per plot for each BC_3_F_6_ progeny. Totally, 192 seeds were sowed per plot. The development of the mapping populations and the screening of recombinants are depicted in Additional file 1: Figure S1.

### Disease scoring in the field

The fine-mapping populations were planted in Baoshan and infected naturally with causal fungus *Cercospora zeina* (*Cz*) [23]. We scored GLS symptoms three times at intervals of 1 week, beginning 2 weeks after pollination. The number and size of disease spots on the leaves of the entire plant were used to evaluate GLS severity. Disease severity was rated using the following scale: 1 (highly resistant), 3 (resistant), 5 (intermediate resistant/susceptible), 7 (susceptible), and 9 (highly susceptible) [22].

### Development of high-density markers in the *qRgls2* region

According to the B73 reference genome v2.0 assembly (B73 RefGen_v2) (http://www.maizesequence.org/index.html), the physical distance of the confidence interval for *qRgls2* is ~110-Mb and covers the whole centromere of chromosome 5. SSR and insertions or deletions (InDels) polymorphism (IDP) markers located in the *qRgls2* region were retrieved from the Maize Genetics and Genomics Database (http://www.maizegdb.org). Because of the low density of these existing markers, we then developed new markers within the QTL region. We downloaded the sequences of the *qRgls2* region from B73 RefGen_v2 (http://www.maizesequence.org/index.html) and mined possible SSR sequences using SSRHunter1.3 software [24]. Single-copy SSR sequences were obtained after BLASTn comparison with the maize high-throughput genome sequence (HTGS) database, and SSR markers were developed using PRIMER5.0 software [25]. For primer design, we searched for single-copy sequences in the *qRgls2* region based on the B73 genome, and we then amplified those sequences from the genomes of the two parents, Y32 and Q11. PCR products were separately cloned into the pGEM-T vector for sequencing. To confirm that the correct sequences were obtained, we projected the sequenced amplicons on the B73 genome using ContigExpress Project software (http://www.contigexpress.com/index.html). Sequence alignment of the two parents revealed InDels that were then developed into IDP markers. Single-nucleotide polymorphisms (SNPs) were used to develop SNP markers. Finally, to ensure that markers were located in the *qRgls2* region, we ran a BLAST analysis on the Gramene website (http://www.maizesequence.org/index.html). For SSR and IDP markers, PCR products were analyzed electrophoretically on a 2% agarose gel or 6% polyacrylamide gel. For SNP markers, PCR products were cloned into a pGEM-T vector and sequenced to determine sequence variation.

### Estimation of the genetic effect of *qRgls2*

Sequential fine-mapping of *qRgls2* was carried out using recombinants and their progeny. The progeny derived from a given recombinant were divided into two genotypes based on their sequences at the *qRgls2* region: the heterozygous Q11/Y32 genotype and the homozygous Q11/Q11 genotype. The disease scales 1 and 3 were classified as resistant, scales 5, 7, and 9 as susceptible. The resistance percentage for each genotype was an estimate of the proportion of resistant plants within the population. The relative difference in the resistance percentage between two genotypes represents the genetic contribution of the introgression region to GLS disease resistance.

### A statistical model for declaration of *qRgls2*

A linear regression model *y*_*i*_ *=* α + β*x*_*i*_ *+* ε_*i*_ was used to test whether a significant association exists between the disease scales and the genotypes in the recombinant-derived progeny. In the backcross population, variable *x*_*i*_ represents the genotype of the donor segment, *x*_*i*_ = 1, when the marker genotype is *Aa* (heterozygous Q11/Y32), or *x*_*i*_ = 0, when the marker genotype is *aa* (homozygous Q11/Q11)*. Y*_*i*_ represents the phenotypic value for the *i*^*th*^ individual, α is an intercept, β is the regression coefficient for *y*_*i*_ on *x*_*i*_, and *ε*_*i*_ is random error [26].

The significance of the regression coefficient was evaluated using the *t*-test. A *P*-value of ≤0.05 indicated the presence of a significant association, and the donor segment covered the *qRgls2* locus. In contrast, a *P*-value of **>**0.05 revealed no significant correlation, and the donor did not contain the *qRgls2* locus. The statistical analysis was run with R 2.15.3 software (http://cran.r-project.org/).

## Results

### Development of high-density markers in the *qRgls2* region

In the initial QTL mapping, *qRgls2* was mapped to a large chromosomal region because of the small mapping population and low-density markers. High-resolution mapping of *qRgls2* is essential for the isolation of genes involved in GLS resistance. We thus mined SSRs in the *qRgls2* region based on the B73 reference genome and identified 826 single/low-copy SSRs. Based on their flanking regions, we designed 826 primer pairs to amplify both parental lines Y32 and Q11. Polymorphic PCR products were observed in 67 out of 826 primer pairs. Of these 67 SSR markers, only 18 were selected to saturate the *qRgls2* region (Table 1), as the other 49 SSR markers were tightly linked to these 18 markers. To develop IDP and SNP markers, we searched B73 reference genome and selected 144 single/low-copy segments in the *qRgls2* region to design primers. These primers were used to amplify the two parental genome DNA and the amplicons were sequenced. Based on sequence variations between Y32 and Q11, we developed 15 IDP markers and one SNP marker. Finally, in the light of amplification efficiency and physical location, 4 of the 15 IDP markers and one SNP marker were used to saturate the candidate interval (Table 1).

**Table 1:** **Newly developed molecular markers in the** ***qRgls2*** **region on chromosome 5**

**Location** ^*^ **(Mb)**	**Marker**	**Forward primer (5′–3′)**	**Reverse primer (5′–3′)**	**Annealing temperature (°C)**	**Type**
67.05	G414	TGCTTCCAAACTCCTCCCTA	GCCTTGAGGGTCACCTTTC	60	SSR
67.51	G520	CACACCACACCAATGCAAAT	CACAGCCATGTTCAGGTCAG	60	SSR
69.40	B96	CCCTGGGCGCAAAGCAAAGG	TCAGACGGTAGTGCAAGGCACC	58	SSR
70.80	G386	CAGCACCCTGCTGGTTATTT	GCGGGTTGAAACCGTAGTAG	58	SSR
70.90	IDP36	TCCTCCTGGCAGTCTAGGAA	TCCGTTTTGTTCTGTTGTGC	60	IDP
71.40	G350	ACCTCTTCGACGCAACACTC	CGTCGATGAACCTCCGTAGA	60	SSR
72.70	G366	GCCTGGAAGCTCGTAGTTGT	GTCCAGTCCGTCCCATAAAA	60	SSR
73.05	G346	CACAGAAGCGTTTCCTTCG	GCTCTGGCTCTGGTTCTAGC	58	SSR
73.11	DD3	GTGTTTCGCCTCTGGATTTC	AAAAACTGCGTTGCCAGTCT	58	IDP
74.05	Q22	GGTGCTCCATTGATTGACCT	CGCCCTGTTCTTATTTGCTC	58	IDP
74.1	DD11	GGAAACAATGGCACACTTCA	GCTTGCATTAGGCTGTTCCT	58	SNP
74.50	G286	TGTCGTCGTTCCATTACGAG	CGGTTTCCGAAAATGAAGAG	60	SSR
74.70	IDP41	TGAAGGCTCCAGCTAATGGT	CCGAGGCACGATAAACATCT	60	IDP
80.19	G241	GATATGGAGGCCCTCTCTCC	ATGATCTCGGTGGTTTCAGG	58	SSR
80.41	G51-1	ATGCACTGATGGGGAGTGAT	TGTTCTCTGAGCACCAGACG	60	SSR
80.83	G206	ATCGGCAGATAAAAGCAGGA	CGGGATAAGGGAGGATGATT	58	SSR
83.08	G192	TTGATGGGCTTAACATTGTCC	TTCGGTTAGGGTGGATTGAG	60	SSR
85.87	G64	GGAAAAGGGAATGGATGGAT	GGAAGGATCAAGGGAAAGGA	58	SSR
95.87	L19	AACTCTGGACTCGCTAGGCA	CCGGATGAAGCTAACTGCA	58	SSR
131.46	xl57	CCGCTCCGCGTATAAAGTAG	CTCTGAAGCCAGGACGGTAG	60	SSR
138.12	G5	CAATGACTTCCGCACCAGTA	CTCGTTGCCGGTCTCTATGT	58	SSR
146.08	35-1	CTCCTTGTTTGGGCCTAATG	TAGGATAAGAGCCCGTGAGC	60	SSR
162.00	xl12	TCCTCCCTCCCTTGATGAAT	CAGGGAGGCTCAGTAATGGA	60	SSR

*Location: The physical location according to B73 RefGen_v2.

### Fine-mapping of *qRgls2*

*qRgls2* was mapped to an interval of ~110-Mb, flanked by the markers umc1784 and umc1171 (Additional file 2: Figure S2) in the initial QTL mapping [22]. It explained 18.9–23.9% of the total phenotypic variation in the mapping population [22]. We conducted a one-way analysis of variance (ANOVA) in the F_2:3_ families using the *qRgls2*-tagged marker G386 (Additional file 3: Table S1). The homozygous Y32/Y32 or heterozygous Q11/Y32 genotype showed a lower disease scale than the homozygous Q11/Q11 genotype, indicating the presence of *qRgls2* in the mapped region (Additional file 3: Table S1). Therefore, we conducted sequential fine-mapping of *qRgls2* from 2011, 2012, and 2013.

**Figure 2:**
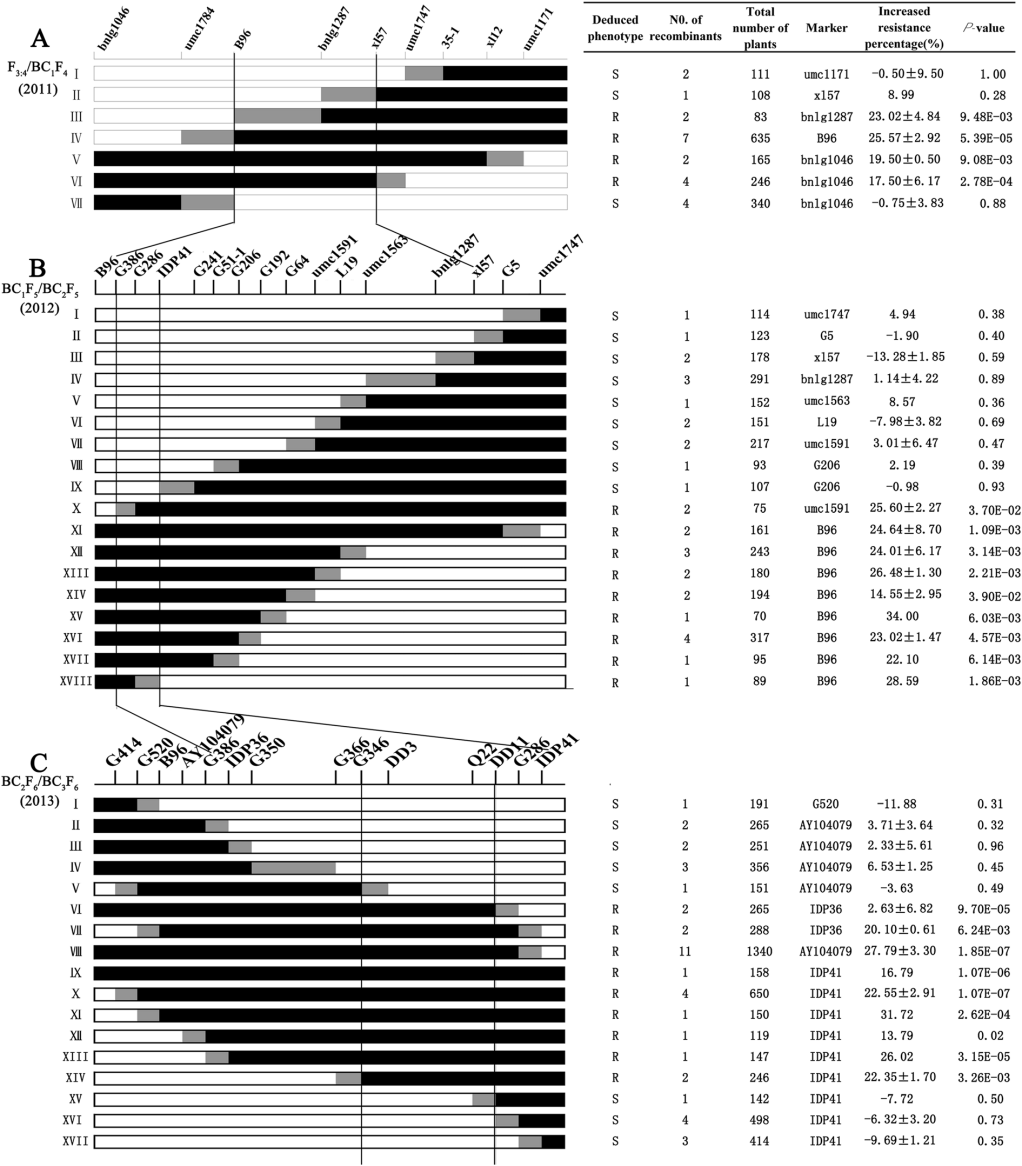
**Sequential fine-mapping of the major QTL** ***qRgls2*** **in recombinant maize cultivated from 2011 to 2013.** The 22 F_4_ **(A)**, 32 BC_1_F_5_ **(B)**, and 42 BC_2_F_6_ **(C)** recombinants were classified into 7, 18, and 17 types, respectively. The genomic architecture for each type is depicted as black, white, and gray rectangles, representing heterozygous Q11/Y32, homozygous Q11/Q11, and mixed regions (in mixed regions recombination occurs, but the exact breakpoint is uncertain), respectively. Table on the right: Increased resistance percentage is defined as the difference in resistance percentage between genotypes Q11/Y32 and Q11/Q11 that resulted from the donor region of Y32; The total number of plants refers to all progeny of a given recombinant type; List of markers used to detect the presence/absence of the donor region; A *P*-value of ≤0.05 indicates the regression coefficient between genotype and disease scale is significant within the progeny derived from a given recombinant type. This suggests the presence of *qRgls2* on the donor region, and the parental recombinant was deduced to be GLS resistant (R). A *P*-value of >0.05 indicates that no significant correlation between genotype and disease scale is present, suggesting that *qRgls2* is absent from the donor region of the parental recombinant, which is therefore marked as GLS susceptible (S). Analysis of both the deduced phenotype and the donor region for all recombinants enabled us to narrow *qRgls2* from an ~110-Mb to an ~1-Mb region, flanked by the markers G346 and DD11.

A total of 22 recombinants screened from the 1,258 progeny of 52 F_3:4_ families were backcrossed to Q11 to generate 22 corresponding BC_1_F_4_ progeny. The sizes of their donor regions were estimated by genotyping at nine markers, including four newly developed markers (B96, xl57, 35-1, and xl12) (Figure 2A). In the summer of 2011, 1,688 BC_1_F_4_ individuals were planted (Figure 2A). All BC_1_F_4_ plants were scored for GLS disease severity and genotyped at the introgression region. The presence of a significant correlation (*P* ≤ 0.05) between genotypes and disease scales indicated that the introgression segment covered the *qRgls2* locus.

For a given F_3:4_-derived BC_1_F_4_ progeny, there are two genotypes, Q11/Q11 and Q11/Y32, at the introgression segment. The recombinants were considered to have the same genotype if they shared the same introgression segment. Thus, the 22 F_3:4_ recombinants were classified into seven types (types I, II, III, IV, V, VI, and VII). Types I and II carried the introgression segments downstream of the umc1747 and bnlg1287 markers, respectively, whereas type VII carried the introgression segments upstream of B96. No significant correlation between genotype and disease scale was detected in the BC_1_F_4_ progeny for each of these three types (*P >* 0.05), indicating that *qRgls2* was absent in their introgression segments. In contrast, types III and IV carried the introgression segments downstream of B96 and umc1784, respectively, and types V and VI had the introgression segments upstream of umc1171 and umc1747, respectively. The regression coefficients between genotype and disease scale were significant (*P* < 0.01) for types III, IV, V, and VI, implying the presence of the resistance QTL, *qRgls2*, in their introgression segments. Thus the *qRgls2* region could be narrowed to an interval between the markers B96 and xl57 (Figure 2A) with a physical distance of ~62-Mb according to B73 RefGen_v2.

Similar analysis was conducted in the BC_1_F_5_-derived 2,850 BC_2_F_5_ progeny in 2012. Sixteen markers were used to genotype 32 BC_1_F_5_ recombinants and to group them into 18 types. Of these, 12 (G386, G286, IDP41, G241, G51-1, G206, G192, G64, umc1591, L19, umc1563, and bnlg1287) were located between B96 and xl57 and two (G5 and umc1747) were located downstream of xl57. Within the mapped *qRgls2* region, types I–IX had downstream introgression segments that did not carry the resistance QTL, *qRgls2* (*P >* 0.05). Type IX had the longest introgression segments of these, indicating that *qRgls2* was not present downstream of G241. Types X to XVIII carried the introgression segments that harbored the resistance QTL, *qRgls2* (*P* < 0.05). Type XVIII carried the introgression segment upstream of IDP41, suggesting the presence of *qRgls2* in the introgression segment upstream of IDP41. The findings from types IX and XVIII thus restrict the right border of *qRgls2* to IDP41. Type X had the closest crossing-over point to the left of the *qRgls2* locus, thus restricting the left border of *qRgls2* to G386. In summary, these three types had the closest recombination breakpoints to *qRgls2* and restricted *qRgls2* to an interval of ~3.9-Mb between markers G386 and IDP41 (Figure 2B).

In 2013, a total of 5,631 BC_3_F_6_ plants derived from 42 BC_2_F_6_ recombinants were planted. Moreover, a total of 14 markers, ten (G386, IDP36, G350, G366, G346, DD3, Q22, DD11, G286, and IDP41) within the *qRgls2* region and four (G414, G520, B96, and AY104079) located upstream of G386, were used to genotype 42 BC_2_F_6_ recombinants and to group them into 17 types. Within the newly mapped *qRgls2* region, types I to V and types XV to XVII did not have *qRgls2* in their introgression segments (*P* > 0.05), suggesting *qRgls2* was present between the left marker G346 and the right marker DD11. Types VI to XIV had the resistance QTL *qRgls2* in their introgression segments (*P* < 0.05) and thus restricted *qRgls2* to the G366 and G286 interval. These types, with and without *qRgls2*, unanimously mapped *qRgls2* into the G346/DD11 interval with the physical distance of ~1-Mb according to the B73 RefGen_v2 (Figure 2C).

### Genetic contribution of *qRgls2* to GLS resistance

**Figure 3:**
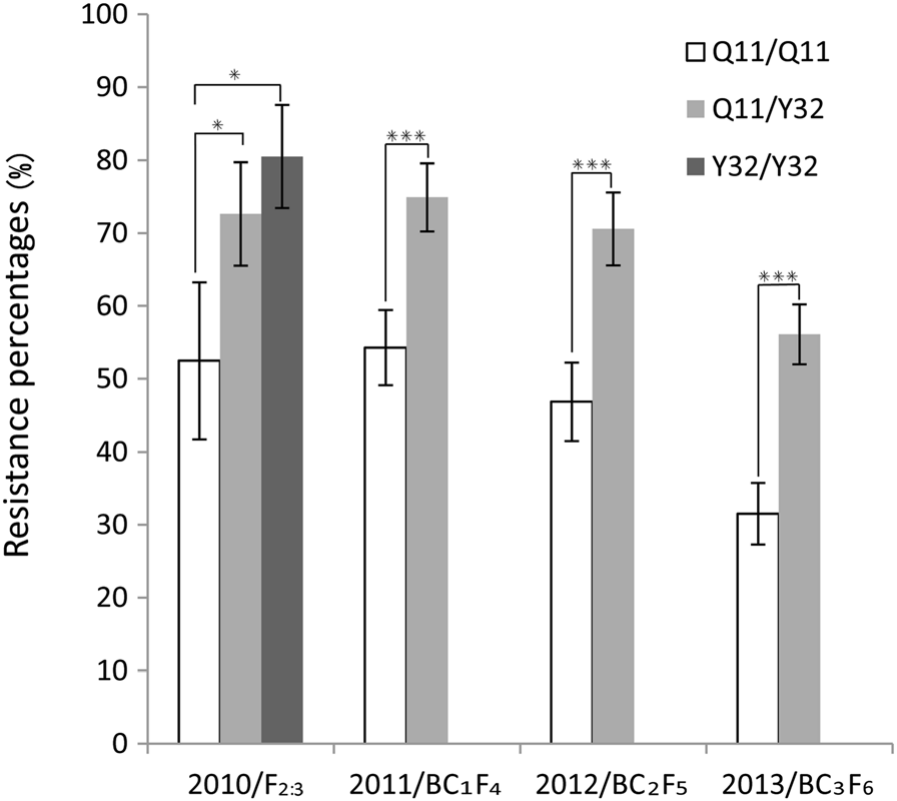
**Estimation of the genetic effect of** ***qRgls2*** **on GLS resistance.** Resistance percentages of different genotypes at *qRgls2* in the F_2:3_, BC_1_F_4_, BC_2_F_5_, and BC_3_F_6_ populations are shown. The *P-*value of the resistance percentage between the genotypes Q11/Q11 and Q11/Y32 from 2010 to 2013 was 0.04, 2.39E-08, 5.57E-08, 4.02E-13, respectively. The *P*-value between the genotypes Q11/Q11 and Y32/Y32 was 0.02 in 2010.

The genetic effect of *qRgls2* was estimated in F_2:3_, BC_1_F_4_, BC_2_F_5_, and BC_3_F_6_ populations, respectively. As expected, plants carrying the *qRgls2* regions showed higher GLS resistance than those without *qRgls2*. In F_2:3_ families, the percentages of resistant individuals were estimated to be 52.5%, 72.6%, and 80.5% for Q11/Q11, Q11/Y32, and Y32/Y32 genotypes, respectively (Figure 3). The resistance percentage in the BC_1_F_4_ population in 2011 was 74.9% in plants with *qRgls2* versus 54.3% in plants without *qRgls2* (Figure 3). Similarly, phenotyping of the BC_2_F_5_ population in 2012 suggested consistent resistance improvement associated with *qRgls2* (70.6% in plants carrying the *qRgls2* segments versus 46.9% in plants lacking the *qRgls2* segments; Figure 3). Finally, in the BC_3_F_6_ population, individuals carrying the QTL regions showed a resistance percentage of 56.1%, whereas individuals lacking the QTL segments only showed 31.5% disease resistance (Figure 3). These results, which were derived from multiple populations over many years, indicated *qRgls2* enhances GLS resistance by 20.6 to 24.6% and that this genetic effect is passed on to subsequent generations.

### Genomic architecture and gene discovery in the *qRgls2* region

The genomic sequence between G346 and DD11 was retrieved from B73 RefGen_v2 (http://www.maizesequence.org/index.html). This region is predicted to contain 15 genes according to the 5b.60 annotation of the maize B73 genome v2 (http://www.maizesequence.org/index.html) (Table 2).

**Table 2:** **Predicted genes in the mapped** ***qRgls2*** **interval and syntenic genes in maize and rice**

**Fine-mapped** ***qRgls2*** **on maize Chr. 5**		**Syntenic gene on Maize Chr. 4**	**Rice**	
**Gene ID**	**Predicted function**		**Syntenic gene**	**Predicted function**
GRMZM2G030013	KH domain–containing protein participates in RNA binding in post-transcription	NA	Os02t0125500	KH domain–containing protein
GRMZM2G477236	lil3 protein, a light harvesting–like protein, plays an essential role in chlorophyll and tocopherol biosynthesis	GRMZM2G027640	Os02t0125700	lil3 protein
GRMZM2G175137	RNA polymerase II transcription factor B subunit 4	GRMZM2G027209	NA	NA
GRMZM2G099827	Microtubule-associated protein with anther-specific expression	GRMZM2G027187	NA	NA
GRMZM5G868966	Unknown	GRMZM5G820374	NA	NA
GRMZM2G157068	Protein kinase	GRMZM2G053868	Os02t0126400	Protein kinase
GRMZM2G456088	Pentatricopeptide repeat (PPR) superfamily protein, participates in post-transcriptional processes in plastids	AC216235.2_FGT010	Os02t0126500	PPR domain–containing protein
GRMZM2G157046	Leaf-expressed protein with unknown function	GRMZM2G053830	Os02t0126600	Unknown
GRMZM2G157026	Golgi SNARE 12 protein	GRMZM5G838961	Os02t0126800	Golgi SNAP receptor complex member
GRMZM2G156983	Phosphatidate cytidylyltransferase	GRMZM2G053711	NA	NA
AC189771.3_FG001	Anther-specific expression with unknown function	NA	NA	NA
GRMZM2G009065	Inflorescence-expressed gene with unknown function	NA	NA	NA
GRMZM2G039254	PPR superfamily protein	NA	Os02t0127600	PPR domain–containing protein
GRMZM2G038791	Ribose-phosphate pyrophosphokinase	NA	Os02t0127700	Ribose-phosphate pyrophosphokinase
GRMZM2G153178	Unknown	NA	Os02t0128100	Unknown

NA: Not available; Chr*.*:Chromosome.

**Figure 4:**
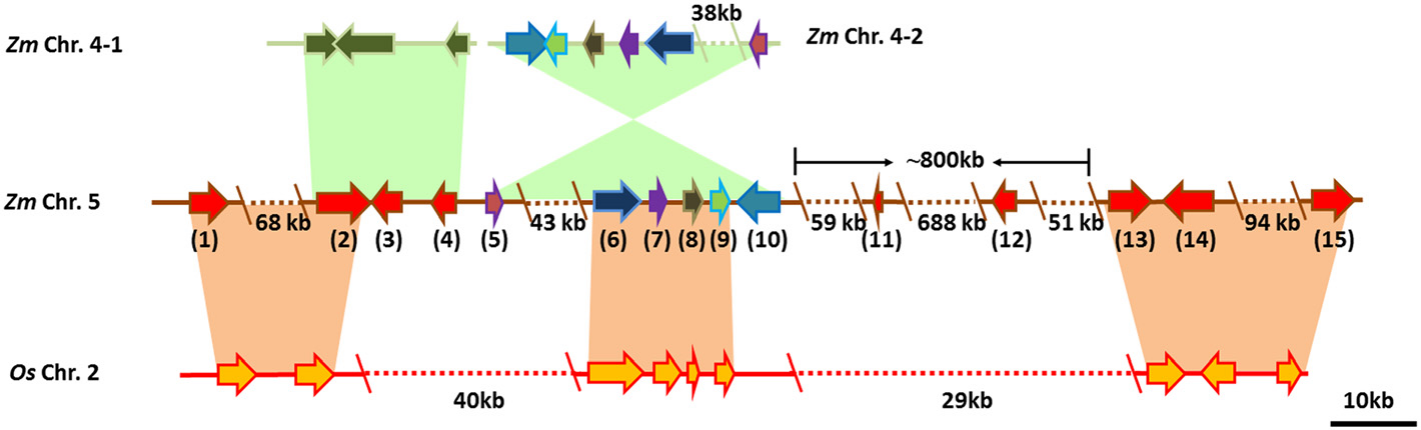
**Genomic architecture of the** ***qRgls2*** **region and its syntenic blocks in maize and rice.** Maize chromosome 4 has two syntenic blocks: *Zm* Chr. 4-1 ranges from 238,744,350 to 238,761,826 bp (~17.5 kb), and *Zm* Chr. 4-2 ranges from 13,562,579 to 13,631,290 bp (~68.7 kb). In rice, the syntenic block *Os* Chr.2 is located on chromosome 2, from 1,334,563 to 1,456,346 bp (~121 kb). The syntenic blocks in maize are depicted as green zones; syntenic blocks between maize and rice are depicted as pink zones. The arrows indicate genes and their orientations; the dotted lines indicate regions without genes. Each pair of reversely syntenic genes with identical sequence is tinted with the same color. The 15 predicted genes in the *qRgls2* region in chromosome 5 are GRMZM2G030013 (1), GRMZM2G477236 (2), GRMZM2G175137 (3), GRMZM2G099827 (4), GRMZM5G868966 (5), GRMZM2G157068 (6), GRMZM2G456088 (7), GRMZM2G157046 (8), GRMZM2G157026 (9), GRMZM2G156983 (10), AC189771.3_FG001 (11), GRMZM2G009065 (12), GRMZM2G039254 (13), GRMZM2G038791 (14), and GRMZM2G153178 (15). *Zm*: *Zea mays*; *Os*: *Oryza sativa*; Chr.: chromosome.

*qRgls2* is adjacent to the centromere of chromosome 5, and the whole region exhibits very low gene density and recombination frequencies as compared with the average across the whole genome [27]. In eukaryotes, genes often show uneven distribution along the chromosomes, and they organize in clusters with different gene densities and sizes [28]. This characteristic is also seen in the *qRgls2* region. The 15 predicted genes are unequally scattered throughout the mapping region. Ten genes are clustered on the left end, three genes are located at the right end, and only two genes are located in the middle portion, which occupies ~800-kb and ranges from 73,235,500 to 74,036,000 bp based on the B73 RefGen_v2 (Figure 4). We analyzed the sequence of this 800-kb gene-desert region using the RepeatMasker (http://www.repeatmasker.org) and found that 92.79% of the sequence consists of transposable elements (90.06% retroelements, 2.59% transposons, and 0.14% unclassified; Additional file 4: Table S2). This percentage is higher than that across the whole maize genome (~85%) [27].

We then searched for duplicated genomic fragments using the Plant Genome Duplication Database (http://chibba.agtec.uga.edu/duplication) and found some genes in the *qRgls2* interval that share high synteny with rice genes located on chromosome 2 (Figure 4). Moreover, we detected two syntenic blocks in maize that are present separately on the short and long arms of chromosome 4 [29]. The one (*Zm* Chr. 4-1) present on the long arm is a part of the large duplicated region between chromosomes 4 and 5, which is assumed to originate from whole-genome duplication followed by genome rearrangement from tetraploid to diploid [30]. The gene content and order within this syntenic block are well conserved, and only a few sequence variations were observed between the coding regions of homologous genes (Figure 4). Intriguingly, the other syntenic block (*Zm* Chr. 4-2), which is located on the short arm, shows perfect reverse collinearity, with the coding sequences of six genes being identical in reverse order in two collinear blocks (Figure 4, Additional file 5: Data Set 1). This inverted synteny may derive from a recent genomic duplication and rearrangement, which took place long after the ancient whole-genome duplication.

## Discussion

GLS resistance is a quantitatively inherited trait and thus hinders breeding of resistant maize varieties [14]. Host resistance is the most cost-effective and efficient way to control GLS disease [8]. Therefore, discovery of a resistance QTL and introgression into elite inbred lines via marker-assisted selection (MAS) would greatly increase GLS resistance [18]. The *qRgls2* locus overlaps with previously reported QTLs [8,20], implying the *qRgls2* region may exist in other mapping populations. Moreover, we showed that the *qRgls2* locus is heritable and could stably enhance resistance percentages by >20% across multiple generations. These findings suggest that *qRgls2* could be used to improve maize resistance to GLS, and high-density markers around the *qRgls2* region will be useful for MAS.

Because of the low density of publicly available markers around *qRgls2*, we retrieved single/low-copy sequences to develop either SSR or IDP or SNP markers to saturate the *qRgls2* region. This strategy, however, turned out to be inefficient. Only a small fraction of the designed primers could be converted into markers. In the future, using genome sequences from diverse maize inbred lines, we can compare *in silico* the mined single/low-copy sequences, for instance between B73 and Mo17, and select those with sequence variations.

Sequential recombinant-derived progeny testing is a powerful method for fine-mapping of resistance QTLs [31]. This strategy can be modified to accommodate different situations. In the current study, we evaluated GLS resistance during the growing season in Baoshan in advanced backcross populations. However, it was impossible for us to produce backcross populations in Baoshan because of severe infections of the recurrent parent line Q11. Therefore, all individuals were selfed, and the resulting self-progeny of newly identified recombinants were grown in winter nurseries (Jinghong or Sanya) where no GLS was prevalent. Heterozygous individuals were then selected and backcrossed to Q11. If the recombinant-derived progeny segregated at the *qRgls2* region, we were able to estimate the relative difference of GLS resistance between two genotypes and to fine-map the *qRgls2* locus. In addition, the advanced backcross progeny shared similar genetic backgrounds with very low background noise [31]. This is very helpful for accurately estimating the genetic contribution of the donor region of a given recombinant to GLS resistance. During screening of recombinants, we were aware that the recombination frequency seems to increase in the *qRgls2* region as backcrossing advanced. The genetic distance per Mb was estimated to be 1.59E-4, 3.06E-4, and 3.78E-3 in F_3:4_, BC_1_F_4:5_ and BC_2_F_5:6_ populations, respectively. We hope to obtain some key recombinants in the *qRgls2* locus when screening more advanced populations. The key recombinants in or around the *qRgls2* locus is critical to narrow a QTL to a QTG (quantitative trait gene), or even to a QTN (quantitative trait nucleotide) for *qRgls2*.

There are 15 functional genes in *qRgls2* region according to the 5b.60 annotation of the maize B73 genome v2. We also evaluated B73 for its resistance to GLS in Boshan and found B73 is highly susceptible to GLS. Thus, none of the 15 genes is likely to be the candidate for *qRgls2*. The resistance gene in the Y32 may be a resistance allele to one of the 15 predicted genes or totally a new gene that is absent in B73. Fortunately, we have constructed the Y32 bacterial artificial chromosome (BAC) library and obtained the positive BAC clones covering the *qRgls2* region. Sequence analysis of the *qRgls2* region of Y32 could reveal all candidate genes, including the resistance gene underlying *qRgls2.*

The ratio of genetic to physical distance in the mapped *qRgls2* region is only ~0.24 cM/Mb, which is much lower than the average ratio of 2.1 cM/Mb across the entire maize genome [32]. Chromosomal recombination occurs more frequently at chromosomal ends as compared with centromere regions in maize [27]. Additionally, gene density is much higher at chromosomal ends than in the centromere regions [28]. The *qRgls2* region is located in the centromere region of chromosome 5 and is characterized by both low gene density (~73 kb/gene) and a low recombination frequency. Furthermore, the 15 predicted genes are unequally distributed in the mapped region. These findings render it very difficult to further screen the key recombinants for fine-mapping, although we continue to expand the mapping population. The same situation was reported for the cloning of *Ghd7*, which is involved in the regulation of the heading date in rice [33]. The candidate gene was mapped to the final 0.31-cM interval in the centromere region of chromosome 7, corresponding to the physical distance of 2,284 kb. Gene annotation identified the candidate gene for *Ghd7*, which was finally cloned via functional complementation [33]. It seems that several approaches are required to clone the genes in the centromeric region. Apart from continued fine-mapping, we will also use transcriptome sequencing and association mapping to confirm the candidate gene for *qRgls2*.

## Conclusions

The development and cultivation of resistant maize hybrids are the most environmentally friendly and cost-effective ways to improve maize resistance to GLS. We identified a major QTL, *qRgls2*, for GLS resistance and narrowed its location from an initial ~110-Mb to a ~1-Mb region. *qRgls2* mainly acted in an additive manner and showed very stable genetic effects across multiple generations. The *qRgls2* region is very close to the centromere of chromosome 5 and is characterized by low and unequal gene density. A total of 15 genes were predicted in the final mapped interval, according to the 5b.60 annotation of the maize B73 genome v2. Our findings provide a solid base for map-based cloning of the GLS resistance gene underlying *qRgls2* in maize. The high-density markers developed around *qRgls2* will be useful in MAS for GLS-resistant breeding.

## Additional files

Additional file 1: Figure S1 Flow chart of QTL identification and fine-mapping. Individual plants from the F_2:3_ families, BC_1_F_4_ progeny, BC_2_F_5_ progeny, and BC_3_F_6_ progeny (as indicated by boxes) were used to evaluate the GLS disease scale. F_2:3_ families were used for QTL identification, and the other populations were used for fine-mapping of the major QTL *qRgls2*. The year, the number of plants/families and locations in each set of experiments was carried out is noted. Additional file 2: Figure S2 Detection of the QTL *qRgls2* across four replicate plots. Logarithm of odds (LOD) profiles (A) and additive genetic effects (B) of the QTL *qRgls2* for GLS resistance. The QTL was detected based on data collected from 161 F_2:3_ families that were grown in Baoshan (two replicates, BS1 and BS2) and Dehong (two replicates, DH1 and DH2) in 2010. Additional file 3: Table S1 Multiple comparisons of disease scales at marker G386. Additional file 4: Table S2 Sequence analysis of gene-desert region using RepeatMasker. Additional file 5: Data Set 1 Alignment of the coding regions for six pairs of syntenic genes. These syntenic genes showed no sequence divergence.

## Abbreviations

GLS: Gray leaf spot
*Czm*: *Cercospora zeae-maydis*
*Cz*: *Cercospora zeina*
SSR: Simple sequence repeat
B73 RefGen_v2: B73 reference genome v2.0 assembly
Mb: Megabase pairs
PCR: Polymerase chain reaction
QTL: Quantitative trait loci
IDP: InDel polymorphism
SNP: Single-nucleotide polymorphisms
MAS: Marker-assisted selection
QTG: Quantitative trait gene
QTN: Quantitative trait nucleotide
BAC: Bacterial artificial chromosome
