# Supplementary figures and images for: High-resolution mapping and characterization of qRgls2, a major quantitative trait locus involved in maize resistance to gray leaf spot
Source: BMC Plant Biol. 2014 Aug 31;14:230. doi: 10.1186/s12870-014-0230-6 (PMC4175277; doi:10.1186/s12870-014-0230-6)

Supplemental Figure 1


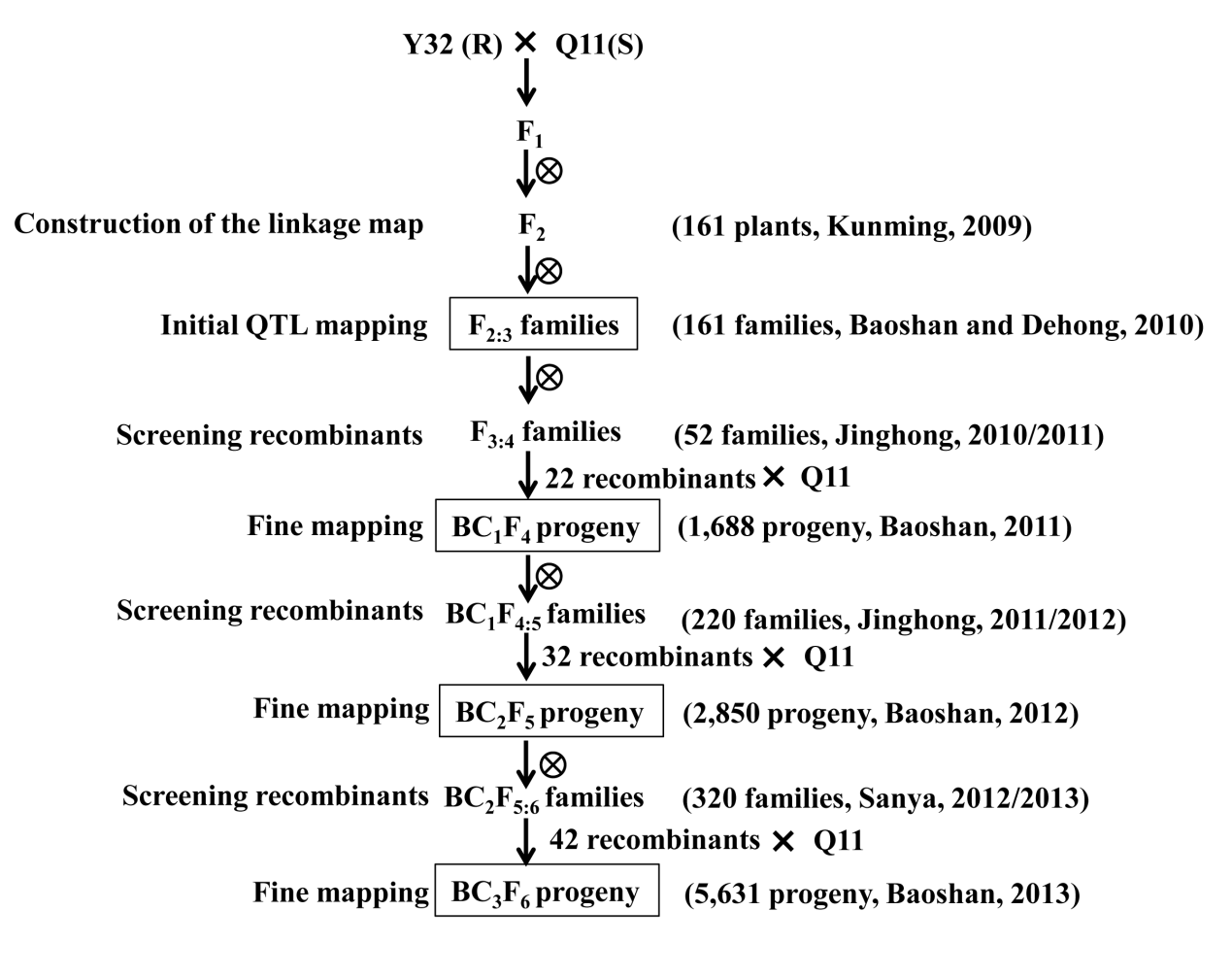

Supplement: Additional file 1: Figure S1 — Flow chart of QTL identification and fine-mapping. Individual plants from the F2:3 families, BC1F4 progeny, BC2F5 progeny, and BC3F6 progeny (as indicated by boxes) were used to evaluate the GLS disease scale. F2:3 families were used for QTL identification, and the other populations were used for fine-mapping of the major QTL qRgls2. The year, the number of plants/families and locations in each set of experiments was carried out is noted. [file 12870_2014_230_MOESM1_ESM.docx]

Supplemental Figure 2


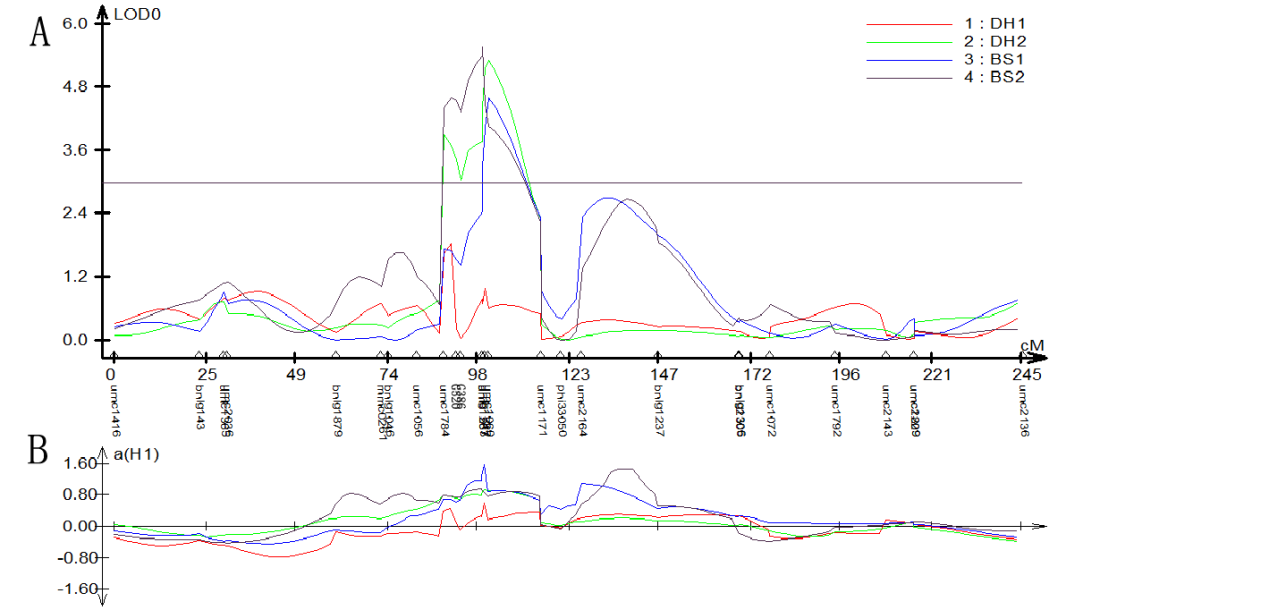

Supplement: Additional file 2: Figure S2 — Detection of the QTL qRgls2 across four replicate plots. Logarithm of odds (LOD) profiles (A) and additive genetic effects (B) of the QTL qRgls2 for GLS resistance. The QTL was detected based on data collected from 161 F2:3 families that were grown in Baoshan (two replicates, BS1 and BS2) and Dehong (two replicates, DH1 and DH2) in 2010. [file 12870_2014_230_MOESM2_ESM.docx]
