# Supplementary material for: High-resolution mapping and characterization of qRgls2, a major quantitative trait locus involved in maize resistance to gray leaf spot
Source: BMC Plant Biol. 2014 Aug 31;14:230. doi: 10.1186/s12870-014-0230-6 (PMC4175277; doi:10.1186/s12870-014-0230-6)
Supplement: Additional file 3: Table S1 — Multiple comparisons of disease scales at marker G386. [file 12870_2014_230_MOESM3_ESM.docx]

**Supplemental Table 1 Multiple comparisons of disease scales at marker G386 (Duncan’s test)**

| Replication^a^ | Genotype | Disease scale (mean) | SE^b^ | **Groups**^c^ |
| --- | --- | --- | --- | --- |
| BS1 | Q11/Q11 | 5.187 | 0.258231 | a |
|  | Q11/Y32 | 4.238 | 0.1630178 | b |
|  | Y32/Y32 | 3.978 | 0.1646016 | b |
| BS2 | Q11/Q11 | 4.213 | 0.3186105 | a |
|  | Q11/Y32 | 3.267 | 0.1486544 | b |
|  | Y32/Y32 | 2.805 | 0.1496424 | b |
| DH1 | Q11/Q11 | 4.107 | 0.2585628 | a |
|  | Q11/Y32 | 3.815 | 0.2055829 | a |
|  | Y32/Y32 | 3.294 | 0.2014456 | a |
| DH2 | Q11/Q11 | 5.596 | 0.2498968 | a |
|  | Q11/Y32 | 4.712 | 0.1669194 | b |
|  | Y32/Y32 | 4.396 | 0.1700461 | b |

^a^BS1 and BS2: two replicates in Baoshan; DH1 and DH2: two replicates in Dehong.

^b^Standard error

^c^Different letters indicate significant differences (P < 0.05) based on Duncan’s multiple range test.
