# Supplementary material for: High-resolution mapping and characterization of qRgls2, a major quantitative trait locus involved in maize resistance to gray leaf spot
Source: BMC Plant Biol. 2014 Aug 31;14:230. doi: 10.1186/s12870-014-0230-6 (PMC4175277; doi:10.1186/s12870-014-0230-6)
Supplement: Additional file 4: Table S2 — Sequence analysis of gene-desert region using RepeatMasker. [file 12870_2014_230_MOESM4_ESM.docx]

**Supplemental Table 2 Sequence analysis of gene-desert region using repeat masker**

| **Element type** | Length occupied | Percentage of sequence |
| --- | --- | --- |
| Retroelements | 691,651 bp | 90.06% |
| SINEs | 165 bp | 0.02% |
| LINEs | 73 bp | 0.01% |
| LTR elements | 691,413 bp | 90.03% |
| Ty1/Copia | 106,222 bp | 13.83% |
| Gypsy/DIRS1 | 585,191 bp | 76.20% |
| DNA transposons | 19,896 bp | 2.59% |
| hobo-Activator | 1,420 bp | 0.18% |
| En-Spm | 5,042 bp | 0.66% |
| MuDR-IS905 | 0 bp | 0.00% |
| Tourist/Harbinger | 91 bp | 0.01% |
| Unclassified | 1,113 bp | 0.14% |
| Total interspersed repeats | 712,660 bp | 92.79% |
| Simple repeats | 1,349 bp | 0.18% |
| Low complexity | 250 bp | 0.03% |
